# Telemedicine in heart failure—more than nice to have?

**DOI:** 10.1007/s12471-018-1202-5

**Published:** 2018-12-10

**Authors:** C. G. M. J. Eurlings, J. J. Boyne, R. A. de Boer, H. P. Brunner-La Rocca

**Affiliations:** 1 Maastricht University Medical Center, Maastricht, The Netherlands; 2 University Medical Center Groningen, Groningen, The Netherlands

**Keywords:** Telemedicine, Heart failure, ehealth and telehealth

## Abstract

Telemedicine in chronic diseases like heart failure is rapidly evolving and has two important goals: improving and individualising care as well as reducing costs. In this paper, we provide a critical and an updated review of the current evidence by discussing the most important trials, meta-analyses and systematic reviews. So far, evidence for the CardioMEMS device is most convincing. Other trials regarding invasive and non-invasive telemonitoring and telephone support show divergent results, but several meta-analyses and systematic reviews uniformly reported a beneficial effect. Voice-over systems and ECG monitoring had neutral results. Lack of direct comparison between different modalities makes it impossible to determine the most effective method. Dutch studies showed predominantly non-significant results, mainly due to underpowered studies or because of a high standard of usual care. There are no conclusive results on cost-effectiveness of telemedicine because of the above shortcomings. The adherence of elderly patients was good in the trials, being essential for the compliance of telemedicine in the entire heart failure population. In the future perspective, telemedicine should be better standardised and evolve to be more than an addition to standard care to improve care and reduce costs.

## Key message

- Telemedicine in heart failure is rapidly evolving.
- Evidence is conflicting, mainly due to a lack of uniform methods/systems.
- Direct comparison between different modalities is lacking which impedes determination of the most effective method.
- Telemedicine should evolve into more than an addition to standard of care.

## Background

Heart failure (HF) is an increasingly prevalent disease, which affects approximately 1–2% of the total population in Europe, despite a tendency towards lower incidence in recent years [1, 2]. The high prevalence is mainly due to the ageing population as the prevalence of HF exponentially increases with age. Not surprisingly, the complexity of the disease is increasing, as well, and the majority of patients with HF suffer from multiple comorbidities [1]. Therefore HF is characterised by high morbidity and mortality, and prognosis improved only slightly despite advances in treatment [3]. The high event rate, particularly repeated hospitalisations, is the main driver of the enormous costs and a substantial reduction in quality of life. In order to prevent these events and to reduce the burden of HF, a multidisciplinary team approach has been advocated [2]. Multiple meta-analyses demonstrated that such an approach indeed reduces the burden of HF [4]. Multidisciplinary treatment not only encompasses optimal therapy of HF, but also involves patient education to improve compliance and self-monitoring by patients. However, such an approach is quite labour intensive, requires many resources and monitoring by patients is often insufficient. Therefore, telemedicine has been suggested to support patients at a distance regarding both education and monitoring and to improve HF care. The implementation of these monitoring tools has been hypothesised to augment medical control of HF to prevent decompensation, to concurrently gain time and resources when compared with traditional care [5] and to maintain a good standard of care in the treatment of HF patients despite the increasing prevalence.

Telemedicine or telehealth are multiform terms embracing the applications of telematics to medicine to enable diagnosis, monitoring and/or treatment remotely by a variety of communication tools, which may include (smart)phones, mobile wireless devices, with or without a video connection, or implantable devices (that are often part of another device such as ICDs or pacemakers [6]). Until recently, digital applications in medicine were restricted to the use of electronic health records, but lately the technological context has notably expanded: the number of existing internet-connected mobile devices has roughly doubled every 5 years [5, 7].

Technology is rapidly evolving. There are a countless number of apps available related to healthcare. New sensors have been developed and data exchange in real-time enables collection of large datasets. Although many issues are not yet resolved (e. g. data safety), expectations are high and there are already healthcare insurers providing reduction in premiums if e‑Health technology is used for prevention or management of diseases. However, the question arises what the exact impact is of this technology on the care in HF, whether it improves quality of life and reduces cardiovascular events, and if it may fulfil its expectations.

## Current evidence

### Implantable devices

**Fig. 1:**
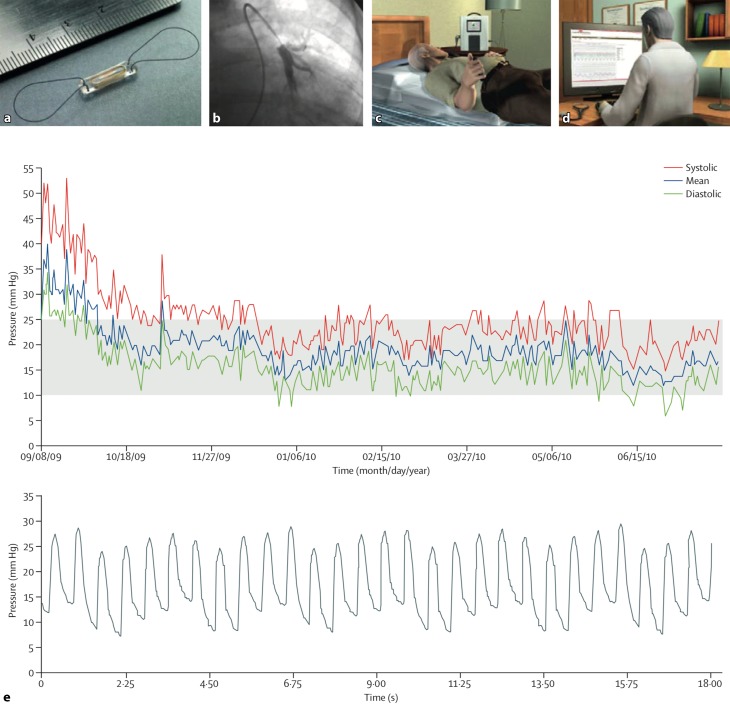
CardioMEMS, implantable haemodynamic monitoring system. **a** CardioMEMS sensor or transmitter. **b** Transcatheter is implanted into a distal branch of the descending pulmonary artery. **c** The patient is instructed to take daily pressure readings from home using the home electronics. **d** Information transmitted from the monitoring system to the database is immediately available to the investigators for review. **e** Transmitted information consists of pressure trend information and individual pulmonary artery pressure waveforms. With permission from Elsevier, original figure from Abraham et al. Lancet. 2011;377:658–66

**Fig. 2:**
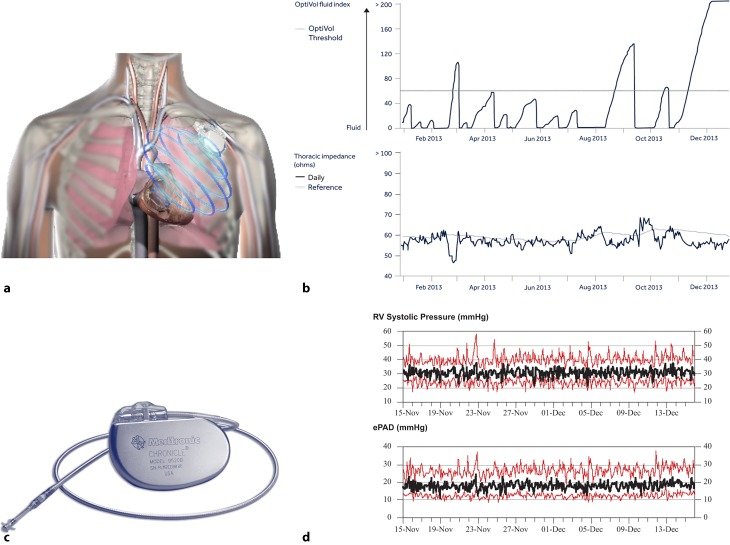
Examples of invasive monitoring. **a** OptiVOL of Medtronic pacemaker/ICD devices. **b** Results presented for OptiVol with the thoracic impedance (ohms) measured and the OptiVol fluid index, resulting from the difference of measured thoracic impedance and reference thoracic impedance, with threshold. As the patient’s lungs become congested, intrathoracic impedance tends to decrease. Similarly, an increase in intrathoracic impedance may indicate the patient’s lungs are becoming more dry. **c** The Chronicle® Implantable Hemodynamic Monitor. **d** Results of Right Ventricle (RV) Systolic Pressure measurements of a sensor on a transvenous lead positioned in the right ventricle and estimated pulmonary artery diastolic (ePAD) pressures. With permission, original figure A/B/C from source: Medtronic Inc. With permission from Elsevier, original figure D from Bourge et al. Am Coll Cardiol. 2008;51:1073–9

So far, the most convincing evidence for a telemonitoring device relates to the implantable CardioMEMS device (Fig. 1; [8]). This device is implanted into the pulmonary artery (PA) and transmits PA pressures to a central service centre. The treating physician receives the results, including the trends over time of these measurements. The physician is advised to react if PA pressure exceeds a certain threshold which suggests congestion, and when it is below the normal range suggesting dehydration. The study was not powered for mortality but showed significant reduction in HF hospitalisation as a result of improved HF management. This effect was maintained in the long term [9]. A comparable rationale was studied in the COMPASS-HF trial. A sensor on a transvenous lead was positioned in the right ventricle (Fig. 2). The primary endpoint rate was reduced by 21%, but this was not statistically significant. There were lower event rates than expected which could make the study underpowered for the primary endpoint [10]. The major limitation of these studies was that the treatment recommendation is very generic, with a plethora of interventions being used (diuretics, vasodilators), at the discretion of the caring physician.

Another form of telemonitoring is part of ICD/CRT devices. Such monitoring has not generated uniform results. The IN-TIME trial reported improved clinical outcomes by using multi-parameter monitoring based on information from an ICD device. By a daily check of several parameters summarised in Tab. 1, a composite clinical score indicating worsening of HF was improved by 37% (odds ratio = 0.63, 95% CI 0.43–0.90) as compared with usual care [11]. The EFFECT study showed a similar effect with a clear reduction of the combined primary endpoint of all-cause mortality and cardiovascular hospitalisation [12]. Encouraging results were also found in the COMMIT-HF trial, a matched cohort study, using different cardiac device brands for the telemonitoring. They observed a long-term effect of significant reduction in mortality (4.9% vs. 22.3%, *p* < 0.0001), but obviously, this was not a randomised trial [13]. In contrast, The OptiLink HF study could not show any beneficial clinical outcome in advanced HF by using fluid status telemedicine alerts (Fig. 2; [14]). Likewise, the positive effects on mortality and cardiac hospitalisation were not supported in a meta-analysis including 11 RCTs consisting of 5,703 patients; there was only a favourable effect on the number of visits, but no effects on hard clinical outcomes and an increase in unscheduled visits [15]. As to whether the differences can be explained by the use of different devices or different interventions is currently unknown. Therefore, no general recommendation to use monitoring information from implantable devices can at present be made.

**Table 1:** Summaries of different international telemedicine studies

Study	Design	*N*	FU in months	Intervention	Primary endpoint	Outcome
TELE-HF (2010)	RCT	1,653	6	TM:	readmission for any reason or death	negative
	2 arms:			telephone based interactive voice-response system. Symptoms and weight daily collected		(difference 0.8% points; 95% CI −4.0–5.6; *p* = 0.75)
	TM vs. UC					
WISH (2010)	RCT	344	12	intervention group:	cardiac rehospitalisation	negative
	2 arms:			electronic scale automatically transmitted weight		(HR 0.90; 95% CI 0.19–1.73; *p* = 0.32)
	TM vs. UC					
CHAMPION (2011)	prospective single blind multicentre trial	550	15	CardioMEMS: wireless implantable haemodynamic monitoring system of pulmonary artery pressures in addition of standard care	HF related hospitalisations	positive
						(HR 0.72; 95% CI 0.60–0.85; *p* = 0.002)
	2 arms:					
	intervention vs. UC					
					no device/system-related complications	positive
						(98.6%; 95% CI 99.3–100.0)
					no pressure-sensor failure	positive
						(100%; 95% CI 99.3–100.0)
TIM-HF (2011)	RCT	710	26	TM: Including daily ECG, blood	mortality	negative
	2 arms:			pressure, body weight		(HR 0.97; 95% CI 0.67–1.41; *p* = 0.87)
	TM + MTS vs. UC					
INH (2012)	open RCT	715	6	HF nurse:	combined: time to death or rehospitalisation	negative
				in hospital contact; teaching materials; UTS; blood pressure/heart rate; up-titrating medication (in cooperation with GPs); weekly contact first, later individualised		
	2 arms:					(HR 1.02; 95% CI 0.81–1.30; *p* = 0.89)
	NTS + UC vs. UC					
CHAT (2013)	RCT	405	12	TeleWatch system	composite of death; HF hospitalisation; withdrawal from study due to worsening HF and improvement of well-being	negative
	2 arms:			follow-up by HF nurses at least monthly regarding: HF clinical status; medical management; social relevant questions		(OR = 1.02; *p* = 0.91)
	UC vs. UC + NTS					
IN-TIME (2014)	RCT	664	12	TM by ICD:	composite of all-cause death; overnight HF hospital admission; change in NYHA class and change patient self-assessment	positive
	2 arms:			Tachyarrhythmia; low % biv-pacing; increase VES; decreased patient activity; abnormal intracardiac electrogram		(OR 0.63; 95% CI 0.43–0.90)
	UC + TM vs. UC					
MCCD (2014)	RCT	204	26	remote monitoring of:	30-day readmission for the first year	positive
	2 arms:			daily weight; blood pressure; heart rate; heart rhythm		
	TM vs. UC					
					all-cause hospitalisation; Average time to hospitalisation; Costs; Mortality and QoL	negative
EFFECT (2015)	prospective, non-randomised trial	987	12	TM by CIED:	combined: all-cause mortality and CV hospitalisations	positive
				study protocol did not mandate any specific device programming and was free to enable the available system integrity and clinical alerts for automatic remote notification		(0.15 vs. 0.27 events/year; incident rate ratio, 0.55; 95% CI, 0.41–0.73; *p* < 0.001)
	2 arms:					
	UC vs. TM + UC					
OptiLink HF (2016)	RCT	1,002	22–23	TM by CIED:	composite of all-cause death and CV hospitalisation	negative
	2 arms:			fluid status alerts; automatically transmitted as inaudible text message to the responsible physician		(HR 0.87; 95% CI 0.62–1.28; *p* = 0.52)
	UC vs. TM + UC					
COMMIT-HF (2017)	observational prospective cohort study	822	36	TM by CIED:	all-cause mortality	positive
				automatic transmission of data from the cardiac device. Daily check of the data by 2 physicians and 2 EP nurses		
						(HR 0.187; 95% CI 0.075–0.467, *p* = 0.0003)
	2 arms:					
	US vs. TM + UC					
TIM-HF2 (2018)	RCT	1,571	Max 13	daily transmission of: bodyweight; blood pressure; heart rate; heart rhythm; SpO_2_; Self-rated health status	percentage of days lost due CV hospitalisations or all-cause death	positive
	2 arms:					(ratio 0.80; 95% CI 0.65–1.00; *p* = 0.0460)
	UC vs. UC + RPM					

*N* number of participants, *FU* follow-upm *RCT* randomised controlled trial, *TM* telemonitoring; *UC* usual care; *NTS* nursing telephone support; *MTS* medical telephone support; *RPM* remote patient management; *CIED* cardiac implantable endovascular device, *VES* ventricular extrasystole, *EP* electrophysiology, *HF* heart failure, *TM* telemonitoring, *OR* odds ratio, *HR* hazard ratio, *CI* confidence interval; *PA* pulmonary artery, *HF* heart failure, *CV* cardiovascular; *QoL* Quality of Life.

### Non-invasive monitoring

**Fig. 3:**
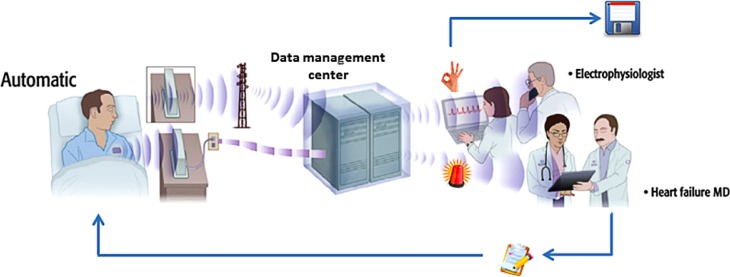
Telemonitoring system for remote monitoring of arrhythmia and heart failure patients. Multi-parameter data acquisition and transmission should be fully automatic with smooth data flow to medical staff/arrhythmia and heart failure monitoring centre. Optimised data workflow: normal data are automatically stored in a patient’s electronic file without further detailed evaluation. Alarm threshold crossing triggers detailed data review and potential medical action. With permission from Oxford University Press, original figure from Varma N, Ricci RP. Eur Heart J. 2013;34:1885–95 and reprinted/adapted figure in Hindricks G, Varma N. Eur Heart J. 2016;37:3164–6

In general, individual trials of telemonitoring/telephone support compared with usual care did not consistently report positive results on the primary endpoints (Tab. 1). The large TELE-HF study did not generate any clinical proof for the use of telemonitoring (utilising a telephone-based interactive voice response system collecting daily information on symptoms and weight) [16]. A voice response system was used without direct contact between healthcare providers, possibly resulting in the low adherence of 14% never users and only about half of the patients using the system more than three times per week [16]. Unfortunately, there was no post-hoc analysis to determine if good adherence resulted in better outcomes. Also, the impact of weight changes for monitoring may be overestimated as it was not demonstrated to be effective as a predictive marker of impending decompensation [17]. This is supported by the negative WISH trial that compared a self-measurement of patients’ weight or by an electronic scales with automatic transmission of the results to the clinic. There was a solid mean of 75% (0–100%) of patient compliance, but there was no significant difference in endpoints between the groups or in subgroups [18]. Also the MCCD trial showed no benefit of telemonitoring despite very good adherence of the participants [19]. Again, the system was mainly based on data transmission with very little direct contact with the patients. Further, the TIM-HF group failed to show a positive effect on the primary endpoint of all-cause mortality or composite endpoints comparing usual care with telemonitoring (Fig. 3), but the study was not sufficiently powered [20]. In addition, the CHAT trial showed mixed results with positive effects on the secondary endpoints of all-cause mortality and all-cause hospitalisation with telecommunication, but not on the primary endpoint in HF patients living in rural areas [21]. Very recently, the large TIM-HF2 study found more days alive outside the hospital with the use of structured remote patient management interventions as compared with usual care (Tab. 1; [22]). Taken together, the inhomogeneity of the methods used, the devices applied, the patients included and the intervention performed together with the lack of sufficient statistical power may explain the mixed findings of individual trials regarding the use of telemonitoring. Moreover, there is a lack of direct comparison between different modalities, making it impossible to determine which may be the most effective method.

However, several recent meta-analyses and systematic reviews reported that the use of telemonitoring may improve outcomes [23–26]. As a consequence of the mixed trials, these meta-analyses included studies with different inclusion criteria. Despite these differences, all meta-analyses reported reduction in mortality and HF-related hospital admissions. In addition, Kruse et al. concluded that telemedicine is effective in customer satisfaction [25] and may increase the sense of security [27]. Also, some but not all studies reported positive effects on quality of life [9, 19, 28]. Still, as many randomised trials were neutral, the recommendation by the ESC HF guidelines is restrictive (i. e. recommendation IIB, level B) [2].

### Dutch studies

In addition to research design, organisation of healthcare may importantly influence outcomes of healthcare interventions as telemonitoring. By comparing only Dutch studies, we attempt to create a certain level of similarity, as organisation of care is comparable in all parts of the Netherlands. The first randomised study including a significant number of Dutch patients was the TEN-HMS study [29], which compared telemonitoring with more traditional HF care as nursing telephone support and usual care. Telemedicine did not differ from nursing telephone support except for prescription of medication, however both had significantly better results compared with usual care for all endpoints (Tab. 2). The Dutch TEHAF study [30] compared the results of using the Health Buddy®, monitoring signs and symptoms, with usual care. HF hospitalisations and visits to the HF clinic decreased, but the primary endpoint of time to first HF hospitalisation was not significantly improved. The IN-TOUCH study compared an ICT-guided disease management support and an ICT-guided support with additional telemedicine [31]. No significant differences in outcome were found, possibly due to the lack of a usual care group. The e‑Vita study, a prospective three-arm study (usual care; usual care plus the heartfailurematters.org website; these two plus an adjusted care pathway with an interactive platform for disease management (e-Vita platform), replacing routine outpatient consultations with HF nurses), could not show any significant benefit [32]. Lastly, an optimised care program using a telehealth application in a pre-post design [33] during a 12-month follow-up found positive effects on most outcomes. Due to the design and the limited study population, the results should be interpreted with caution.

**Table 2:** Summaries of different Dutch telemedicine studies

Study	Design	*N*	FU in months	Intervention	Primary endpoint	Outcome
TEN-HMS study (2005)	RCT	426	14–15	TM:	TM vs. NTS:	negative
	3 arms:			electronic monitoring of weight; blood pressure; single lead ECG	days lost because of death or hospitalisation	(difference −4 days; CI −15–6)
	UC; TM; NTS					
				NTS: (nursing telephone support)	TM, NTS vs. UC:	positive
					days lost because of death or hospitalisation	(difference 6 days; 95% CI 1–11)
TEHAF (2010)	RCT	382	12	Health Buddy:	Time to first hospitalisation	negative
	2 arms:			Monitoring signs & symptoms; Education; Support of self-care		(HR 0.65; 95% CI 0.35–1.17; *p* = 0.151)
	UC; TM					
IN TOUCH (2016)	RCT	177	9	innovative ICT-guided-disease management support combined with TM	composite endpoint of mortality, HF readmission and change in health-related quality of life	negative
	2 arms:					(Mean difference 0.1; 95% CI −0.67–0.82; *p* = 0.39)
	innovative ICT-guided support; Innovative ICT-guided support + TM					
				electronic monitoring of weight; blood pressure; ECG (used in case of start-up or up-titration of beta-blockers)		
e-Vita (2018)	RCT	450	12	heart Failure Matters website	self-care	negative
	3 arms:			care pathway on e‑vita platform		HFM vs. UC mean 72.1 vs. 72.7, and EACP vs. UC 76.1 vs. 72.7, respectively (overall *p* = 0.184)
	UC; UC + HFM website; care pathway + link to HFM website					
Hart Motief Study (2015)	pre-post design	102	12	Motiva telehealth system: providing educational material, reminders of medication and motivational messages	no. of HF-hospitalisations	positive
						(rate ratio 4.1; 95% CI 2.8–6.3; *p* < 0.001)

*N* number of participants, *FU* follow-up, *RCT* randomised controlled trial, *TM* telemonitoring, *UC* usual care, *NTS* nursing telephone support, *MTS* medical telephone support, *CIED* cardiac implantable endovascular device, *HF* heart failure, *TM* telemonitoring. *OR* odds ratio, *HR* hazard ratio, *CI* confidence interval, *PA* pulmonary artery, *HF* heart failure, *CV* cardiovascular´, *QoL* quality of life

Taken together, the Dutch studies follow the line of the overall evidence with mixed results, explained by the low power of the studies. Endpoints mostly focus on mortality and care consumption, yet they were not powered to detect differences. Possibly, the high standard of usual care may have influenced the results. The challenge is to detect the important aspects of the systems and how to integrate the systems into the daily care process.

### Cost-effectiveness

There is limited evidence regarding cost-effectiveness of telemedicine. The reduction of hospitalisation and the increased self-management of patients embodies the potential of cost reduction in healthcare [25]. The incremental cost-effectiveness of the CardioMEMS device is estimated to be $ 13,979 per quality-adjusted life year gained [8]. Klersy et al. describe in their meta-analysis on telemonitoring by cardiac devices a reduction of 44% in hospital visits, without affecting mortality, resulting 15–50% cost saving [15]. In the long term, these interventions were calculated to be cost-neutral [34].

Regarding non-invasive telemonitoring, the effects on costs are even less clear. Blum et al. showed no cost-reduction [19] as there was no positive effect in the study (e. g. readmission/hospitalisation). In contrast, Comín-Colet et al. found a significant reduction in HF and cardiovascular readmission with the use of telemedicine, which resulted in a net decrease in direct hospital costs of € 3,546 per patient per 6 months of follow-up [35]. In the Dutch TEHAF study, the probability of being cost-effective was only 48% (threshold of € 50,000), possibly due to the divergence between participating institutions [36]. Because of the heterogeneity of all the studies, populations and no uniform intervention it is difficult to be conclusive on cost-effectiveness.

## Potential shortcomings and limitations

One of the shortcomings of telemedicine may be that patients need to be able to use modern technology. This may particularly apply to elderly patients, who usually have less exposure to ICT and may, therefore, either be unable or unwilling to use this technology [37]. Still, a recent meta-analysis shows that patients with a mean age of 70 years or more can quickly adopt telehealth, find its use an acceptable part of their healthcare routine and are able to maintain good adherence for at least 12 months with a beneficial effect in reducing the risk of all-cause mortality and HF-related hospitalisations [38]. The same result is shown in a post hoc sub-analysis of a Cochrane analysis [26, 39]. Still, it must be stressed that it is very likely, though not specifically reported, that patients were selected and these findings might not be applicable to all patients with HF. This is in line with a recent finding that participants and non-participants of e‑Health technology in HF differed significantly, particularly regarding age [40]. Nevertheless, these findings are interesting and promising that technology can developed in a way that it is easy to use for a large proportion of HF patients [38].

Another shortcoming is that the influence of reimbursement adopted by the insurance companies is probably significant but not yet tested. It is also unclear if the reimbursement strategy results in a more structured use of telemedicine. Also, the organisation of care may influence the effects of telemedicine. For this reason, the CardioMEMS system will only be reimbursed in the Netherlands within a prospective randomised study to test if the results of the CHAMPION trial also apply to the Dutch healthcare system.

Moreover, it may be argued that the effect of telemedicine may be largest in rural areas where access to good quality healthcare may be more difficult. The current data do not clearly support that notion, but studies did not properly investigate the impact of remoteness of access to care.

Finally, data safety will be an important issue, particularly for next generation devices that may include data from electronic patient records. So far, telemedicine was used mainly as stand alone, limiting data safety issues but also enhanced functionality. Therefore, issues of data safety should be addressed more extensively with further development of (new) devices.

## Future perspectives

There are two main goals of telemedicine in HF: improving care and reducing costs. It is not necessarily required that telemedicine devices must strive to achieve both, but the present and future requirements in healthcare will actually favour devices aiming to do so. It is important to much better define which goals are important to improve outcome, as highlighted above.

Thus far, theoretical considerations have formed the basis for developing telemedicine devices. These included the idea that monitoring patients regarding signs and symptoms, via haemodynamic monitoring or as part of implantable devices such as ICDs (e. g. impedance, heart rate variability, activity levels) would result in a reduction in acute decompensation. This assumption is not sufficiently supported, and it is largely unknown what is required to achieve the best outcome. Best results were achieved with the use of invasive haemodynamic monitoring [8, 9], but this is not applicable to the majority of patients and confirmation in other healthcare systems than initially tested is required [41, 42]. In addition, a similar kind of device use (i. e. ICD/CRT-D devices for remote monitoring) resulted in mixed results [11, 43], which cannot be easily explained. Importantly, the exact action required based on the result of monitoring is left to the care professionals in charge, which obviously may vary significantly. Therefore, there is an urgent need for randomised controlled trials with a clear definition of both monitoring and intervention modalities, as well as collection of comprehensive data from the clinical use of telemedicine devices. Combining such data based on different systems may help define which parts of monitoring and patient education are most effective. However, there is also a great need to sufficiently record and analyse the therapeutic intervention done based on telemedicine systems. So far, there is a lack of such data in sufficiently large patient populations.

Telemedicine has also been advocated to reduce costs in HF care [35], mainly related to reduction in hospitalisation rate. However, there may be also a significant improvement in self-management in HF as well as other chronic diseases [44], possibly resulting in reduction of outpatient visits as shown for another chronic disease [45]. Current systems have limited abilities to foster self-management by patients. Healthcare in Western countries requires a new innovative approach to address chronic diseases such as HF to provide sustainability of care and to limit the excessive costs that may threaten the current system. Thus, changing the approach to care is important, not only regarding adoption and smarter use of modern technology, but also regarding a new vision on both care and health [46]. Therefore, telemedicine should be more than an addition to standard of care. Importantly, chronic diseases usually do not occur in isolation. Most patients with chronic disease have multiple diseases [47]. Future telemedicine devices for HF should consider comorbidities, not only for safety reasons, but to enable real patient self-management that may enable some substitution of traditional care.

## Conclusions

Telemedicine is evolving fast, but lacks solid evidence on clinical outcomes and cost-effectiveness in trials, despite positive meta-analysis. The CardioMEMS device showed the most convincing results. For the future, sufficiently powered trials with clear definition of both monitoring and intervention are urgently needed. Telemedicine should evolve to be more than an addition to standard of care. Only then will telemedicine be more than nice to have.
